# ST08 Altered NF-κB Pathway in Breast Cancer Cells *In Vitro* as Revealed by miRNA-mRNA Analysis and Enhanced the Effect of Cisplatin on Tumour Reduction in EAC Mouse Model

**DOI:** 10.3389/fonc.2022.835027

**Published:** 2022-05-09

**Authors:** Snehal Nirgude, Sagar Desai, Raghunandan Mahadeva, Febina Ravindran, Bibha Choudhary

**Affiliations:** ^1^ Institute of Bioinformatics and Applied Biotechnology, Bengaluru, India; ^2^ Division of Human Genetics, Children’s Hospital of Philadelphia, Philadelphia, PA, United States; ^3^ Manipal Academy of Higher Education, Manipal, India

**Keywords:** integrated transcriptomic approach, synergistic, tumor regression, Curcumin derivatives, apoptosis

## Abstract

ST08 is a novel curcumin derivative that exhibited apoptotic and anti-migratory activity in MDA-MB-231, triple-negative breast cancer cells reported earlier. In this study, we further explored the anticancer properties of ST08. ST08 reduced tumor burden *in vivo* and induced apoptosis through the mitochondrial pathway both *in vitro* and *in vivo*. ST08 potentiated the effect of cisplatin *in vitro* and *in vivo* in mouse EAC breast cancer models with minimal toxicity. ST08 induced alterations in the gene expression were studied by parallel analysis of miRNA and mRNA. 74 differentially expressed miRNA regulated 114 mRNA in triple-negative (MDA-MB-231) cancer cells. Pathway related to the ECM was altered in mesenchymal MDA-MB-231 cells. We constructed a unique miRNA-mRNA interaction network, and one of the pathways regulated by miRNA was NF-κB. Targets of NF-κB like MMP1, PTX3, and MMP2 were downregulated in MDA-MB-231 in response to ST08 treatment. PMA induced cell proliferation was abrogated by ST08 treatment, and no additional cell cytotoxicity was observed when used in combination with IKK-16 indicating ST08 regulation of NF-κB pathway in MDA-MB-231 cells.

## Introduction

Breast cancer is the second most common gynecological malignancy causing death in women (1). It is not a single disease but a group of neoplastic disorders with distinct molecular features. Though surgery, radiation therapy, hormonal therapy, immunotherapy, and targeted therapy are routine treatments, the mortality rate due to breast cancer metastasis is still high (2). Among many cytostatic agents used for chemotherapy, platinum analogs are used primarily for patients with BRCA1 mutations (3). These mutations give rise to defects in the DNA double-strand break repair. Thus, platinum analogs like cisplatin, the interstrand cross-linking agents, lead to apoptotic cell death of dividing cells (4). At least 70% of TNBC (triple-negative breast cancer) patients with a germline BRCA1 mutation develop metastatic disease (4). However, due to adverse side effects and chemo-resistance, the use of platinum-based chemotherapy is limited. Thus, the demand for highly potent and effective drugs continues.

Among several reported natural compounds, Curcumin(1,7-bis(4-hydroxy 3- methoxyphenyl) -1,6-heptadione-3,5-dione or diferuloylmethane), has exhibited its pleiotropic anticancer activity profoundly in a wide array of cancer cells like breast (2, 5), colon (6), lung (7), head and neck (8), leukemia (9), pancreatic (10); affecting multiple pathways. Though curcumin is known for its anticancer properties, the primary limitations like low bioavailability and rapid metabolism (2, 11) open the doors for exploring its derivatives. One such widely studied group of curcumin derivatives is DAPs (diarylidenyl-piperidone). DAPs exhibit properties similar to curcumin, with better potency and multidrug resistance reverting property (5). ST08 is one such DAP whose anticancer properties have been explored in greater detail in this study. Like ST09 (2) and ST06 (12), reported from our lab, ST08 has exhibited its efficacy in the nanomolar range (5). We have used *in vitro, in vivo*, and transcriptomic approaches to evaluate molecular mechanisms modulated by ST08 to bring about phenotypic manifestations such as apoptotic cell death and tumor regression *in vivo*.

Curcumin and its derivative, like EF24, are known to regulate the epigenome by micro-RNAs(miRNA) expression (13–15). miRNAs,~22nt short non-coding RNAs, regulate gene expression by either blocking mRNA translation or degrading the mRNA using sequence complementarity (16, 17). In cancer, dysregulation of miRNAs regulating various biological processes related to oncogenesis has been studied (18, 19). One of the common pathways deregulated in cancer-driving oncogenesis is NF-κB. NF-κBis an inducible transcription factor(TF) that regulates several cellular processes (20) that drives breast cancer (21). NF-κBhas a significant role in angiogenic neovascularization, extracellular matrix(ECM) organization, the epithelial-mesenchymal transition (EMT), cancer cell stemness which ultimately leads to cancer cell resistance, early relapse, and poor survival (21). Curcumin is known to regulate NF-κB signaling in various cancers like liver (22), cervical cancer (23), oral cancer (24), renal cancer (25), including breast cancer (26). Curcumin derivatives like EF24 (27), EF31 (28), MS65 (29) are also known to target NF-κBwhen used in the micromolar range. However, this study shows that ST08 mediated the regulation of NF-κB when used in the nanomolar range.

Integrated RNA-seq and miRNA-seq analysis provide insight into drug-induced alterations in the transcriptome at different levels, including quantifying protein-coding and non-coding gene expression, fusion genes, and alternative splicing. Besides, mechanistic insights of gene expression regulation by miRNA, either by translational control or mRNA degradation, can be assayed. Transcriptomic changes capture common and unique pathways induced by a drug in different cell lines (30). RNA-seq, miRNA-seq analysis helps in unbiased detection of both coding and non-coding novel transcripts and transcripts with low abundance (31). As compared to microarrays, NGS sequencing is more sensitive and accurate due to better discrimination between highly similar sequences (32).

ST08, a recently reported derivatized curcumin from our lab, has been shown to curb migration and invasion by targeting MMP1 (5). Here we further explore the anticancer characteristics of ST08 and show its effect on cell cycle, apoptosis in breast cancer cells MCF7 and MDA-MB-231, and the breast tumor mice model. We show that the combination of ST08 and Cisplatin reduces the tumor volume drastically with no apparent toxicity *in vivo*. We have explored breast cancer cells’ whole transcriptomic response(mRNA and miRNA) upon ST08 treatment. We have integrated the miRNA-seq with mRNA-seq and shown miRNA-mediated regulation of ECM-related pathways in MDA-MB-231 cells. Network analysis using string revealed NF-κBand its downstream partners. In the above network, transcription factors regulating miRNA and miRNA regulating NF-κB were manually integrated. We further validated NF-κB and its downstream targets in TNBC MDA-MB-231 cells post ST08 treatment. We correlated the ST08 induced reversal in gene expression, as observed in normal breast vs. tumor samples from GEPIA (33).

## Materials and Methods

### Cell Culture

MDA-MB-231 and MCF7 were purchased from the National Centre of Cell Culture (NCCS), Pune, Maharashtra, India. MDA-MB-231 cells were grown in Dulbecco’s Modified Eagle’s Medium (DMEM high glucose with L-glutamine; Lonza) and MCF7 in Eagle’s Minimum Essential Medium (EMEM; Lonza supplemented with non-essential amino acids (NEAA) from MP biomedicals). All media were supplemented with heat-inactivated 10% fetal bovine serum (Gibco), 100 IU mg/mL penicillin/streptomycin (Gibco) at 37°C in a humidified atmosphere containing 5% CO2. ST08 was dissolved in DMSO such that all treatments had equal concentrations of dimethyl sulfoxide (DMSO) between 0.1–0.2%. Cisplatin(MP biomedicals, Santa Ana, California, USA) was dissolved in water. Phorbol ester (phorbol 12- myristate 13-acetate; PMA) and IKK-16 were purchased from Tocris Bioscience (Bristol, United Kingdom) and dissolved in DMSO. The structure of ST08 and molecular signatures of each cell line are tabulated in **Table 1** (34).

**Table 1:** Molecular Signatures of the cell lines in the study.

Structure of ST08	Characteristics	MCF7	MDA-MB-231
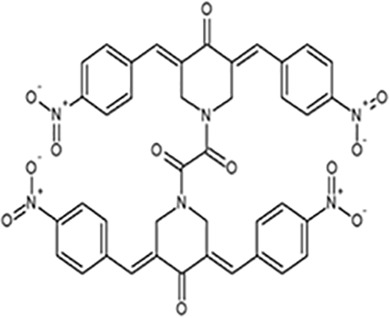	Breast Cancer Subtype (34, 35)	Luminal A	Claudin-low triple negative
	ER,PR, Her2 status (34, 35)	ER+ve,PR+ve,Her2-ve	ER-ve,PR-ve,Her2-ve
	p53 Status (36)	Wild type	Mutant (protein variant p.R280K)
	BRCA1 status (37)	Wild type	Wild type

### Cell Cycle Analysis

Cell cycle analysis was performed in MDA-MB-231, MCF7 cells as described (2, 5, 37). Cells were seeded at a density of 75000 cells/ml and incubated for 24 h at 37°C. After incubation, cells were treated with ST08(0, 20, 40, 60, 80, 100, 120, 150 nM) for 48 h. Cells were harvested by trypsinization, washed, fixed with 80% cold ethanol overnight at -20°C. Then cells were washed incubated with RNase A followed by staining with propidium iodide (PI) (1 μg/ml), incubated at 37°C for 35 min, and readings were acquired in the flow cytometer (Gallios, Beckman Coulter, Miami, FL). A minimum of 10,000 cells/events was acquired per sample, and data were analyzed using Modfit LT free trial version 3.3 available from Verity software house. Experiments were repeated three times, and data were represented along with error bars.

### Phosphatidylserine Externalization Assay

Early apoptotic cells, late apoptotic cells, and necrotic cells were analyzed using Annexin V-FITC/PI staining described earlier (2, 5). Annexin V-FITC binds to phosphatidylserine, which gets translocated from the inner side of the cell membrane to the cell membrane’s outer side during the earlier stage of apoptosis. PI stains both late apoptotic and necrotic cells. MCF7 cells were treated with ST08 (0, 20, 60, 100, 120, 150 nM) for 48 h, stained with Annexin V-FITC for 20 minutes, and PI was added just before analysis examined by flow cytometry (Gallios, Beckman Coulter, Miami, FL). A minimum of 10,000 cells/events was acquired per sample, and data were analyzed. Experiments were repeated a minimum of three independent times, and data were represented with error bars. Every sample was pooled from three independent wells of a 6-well plate.

### JC-1 Mitochondrial Membrane Potential (ΔΨm) Assay

Mitochondrial membrane potential was measured after treatment with ST08, using JC-1 dye. The assay was carried out, as described earlier (2). JC-1 (5,5’,6,6 tetrachloro-1,1’,3,3’-tetraethylbenzimidazol-carbocyanine iodide) is a carbocyanine dye that selectively enters mitochondria and changes reversibly its color from red (J -aggregate, emission at 590 nm) to green (monomeric form, emission at 530 nm) upon a change in mitochondrial membrane potential, that occurs during apoptosis. Briefly, cells were treated with ST08 (0, 20, 40, 60, 80, 100, 120, 150 nM), harvested after 48 h, and incubated with JC-1 dye. The stained cells were then analyzed using a flow cytometer (Gallios, Beckman Coulter, Miami, FL). Cells treated with 2,4-Dinitrophenol (2,4-DNP) served as a positive control. The ratio of cells emitting red to green fluorescence for each concentration was plotted. A minimum of 10,000 cells/events was acquired per sample, and data were analyzed. Experiments were repeated a minimum of three independent times, and data were represented with error bars.

### Immunoblotting

MDA-MB-231, MCF7 cells were incubated with different concentrations of ST08 (0,20,40,75,80,100 nM) for 48h, and western blotting was performed as described (2, 5, 34). The tumor tissues(100mg) from two groups (Control and ST08 treated) were minced using liquid nitrogen. The powdered tissue was sonicated in RIPA buffer, and the supernatant was collected after centrifugation at 12000 rpm, 4°C, 15 min. The supernatant containing protein was quantified using Bradford’s reagent(Biorad), and then immunoblotting was performed (2, 12). The membrane was probed with appropriate primary antibodies involved in apoptosis, such as Apaf1, Cytochrome c,p53 were purchased from Santa Cruz Biotechnology; Bax, PARP, Caspase 3, and Caspase 9 from Cell Signaling Technology; PTX3, MMP1, MMP2, Bcl2 from Cloud clone Corp and NF-κB from Biolegend. The membrane was probed with HRP-conjugated secondary anti-rabbit, anti-mouse antibody (Cell Signaling Technology). The blots were developed with chemiluminescence reagent (Clarity Western ECL blotting substrate, Biorad), and the blot images were captured by the Chemidoc-XRS Biorad gel doc system. The protein band images were quantified using GelQuant.Net, BiochemLab solutions.

### Breast Cancer Mice Tumor Model

The approval for the study was obtained from the Institutional animal ethics committee (Reg. No. 1994/GO/ReBi/S/17/CPCSEA), and all experiments were performed following institutional and national guidelines and regulations of the CPCSEA as described (2, 12). Ehrlich ascites breast adenocarcinoma (EAC) is a spontaneous breast adenocarcinoma model used to screen anticancer drugs for over 4 decades. EAC (1 x 10^6^ cells/animal) was injected to induce solid tumors in the left thigh region of Swiss albino mice. After animals had developed tumor of size ~200 mm3 the animals were segregated in 4 groups: control (n=5) and ST08 treated (n=5), Cisplatin treated (n=5), Cisplatin+ST08 treated(n=5). The treated group animals were then subjected to 10 doses of 20mg/kg of body weight(bd wt) of ST08, 1mg/kg bd wt of cisplatin, and ST08 (10mg/kg body weight)+Cisplatin (1 mg/kg bd wt) intraperitoneally (i.p) every alternate day. The experiment was repeated twice with five animals each for the control, ST08 group. Changes in the tumor size and body weight were observed for 20 days from the day of treatment. Tumor volume was calculated using the formula V = 0.5x a x b^2^, where V is tumor volume, a, b are major and minor tumor diameters.

### Drug Toxicity and Side Effect Assessment on ST08, Cisplatin Treatment

EAC tumor-induced tumor mice in the treatment group were treated with ST08 for 20 days and then were evaluated for drug toxicity. Blood samples were collected from animals from all the groups. Serum was separated, following which drug toxicity biomarkers such as aspartate aminotransferase (AST), alanine aminotransferase (ALT), and BUN were estimated according to the method described by ALT/AST/BUN activity assay kit (Auto span, Span Diagnostics, Bengaluru, India).

### Histological Analysis of Tumor Tissues

Histological evaluation through Haematoxylin-Eosin (HE) staining was done by fixing 20-day old tumor tissues and organs from animals following treatment with the drug treatment in formalin. The tissues were dehydrated, cleared in xylene, then embedded in paraffin, and processed as previously described (2).

### Drug Combination Study by MTT Assay

MTT assay was performed as described earlier (2, 5). Cells were seeded (5000 cells/well) in a 96-well plate in triplicates, incubated for 24 h, and treated with various concentrations of Cisplatin (0,1,5,10,20,50 µM) and ST08 (30,60nM). Cells were incubated with MTT reagent, MP Biomedicals (5 mg/ml) after 48h of incubation of cells with the drugs at 37°C and 5% CO2. Absorbance was measured at 570 nm, and the results shown are from 3 different biological replicates. To understand the effect of the combination of Cisplatin with ST08, the combination index method (38, 39) was used. The combination index is calculated as follows:


$$\text{CI=}\frac{{\text{C}}_{\text{AX}}}{{\text{IC}}_{\text{X,A}}}+\frac{{\text{C}}_{\text{BX}}}{{\text{IC}}_{\text{X,B}}}$$

where C_AX_, and C_BX_ are the concentration of drugs A and B used in combination to achieve x % drugeffect. IC_X,A_ and IC_X,B_ are the concentrations for single agents to achieve the same effect. CI < 1 implies Synergism, CI = 1 is Additive and CI > 1 is Antagonistic effect.

### RNA Preparation and HiSeq2500 Sequencing

Drug Treatment, RNA isolation, and Library preparation: The experiment was performed as described (34). Briefly, 0.75 x 10^5 MDA-MB-231 cells were seeded in each well of a 6-well plate and were treated with 75nM ST08. After 48h of treatment, cells were trypsinized, and cells from three wells having the same treatment were pooled together. After two PBS washes, RNA extraction was done using Trizol Reagent (Ambion) following the manufacturer’s recommendations. RNA concentration and purity were checked using Qubit(Invitrogen, Life Technologies, Carlsbad, California, United States), and its integrity was examined by capillary electrophoresis (Tapestation, Agilent Technologies, Santa Clara, California, United States) to ensure RNA integrity number >9, for a good RNA library preparation. Paired-end RNA-seq libraries were prepared using Illumina TruSeq RNA Library Prep Kit v2.

mRNA library preparation: As described (34), mRNAs were separated using oligo-dT beads and fragmented to 200-250 bp from the total RNA. After synthesizing cDNA, the ends were repaired for blunt ends, and the 3’ ends were adenylated. To the adenylated sites, adapters were linked, and subsequently, PCR amplification of the library was done. After constructing the libraries, their concentrations and insert sizes were detected using Qubit and Agilent Tapestation, respectively. High throughput sequencing was performed using Illumina HiSeq2500 to obtain 100-bp paired-end reads.

miRNA library preparation: RNA isolation was done as mentioned above (34), and RNA sample was given for library preparation. miRNA-library preparation was outsourced to SciGenom Labs, India. In brief, after checking the quality, RIN of RNA, 3’ and 5’ adapters were ligated to the short mature miRNA sequences. After adapter ligation, reverse transcription was done to obtain single stranded cDNAs. The cDNA was then PCR amplified, and the amplicons were run on 8% native PAGE. The gel purification was carried out for ~150bp library, and the libraries’ quality was checked using Tapestation 2200, Agilent. The libraries were then used for pooling and sequencing in Hiseq 2500, Illumina.

### Differential Expression Analysis

mRNA-seq: As described (34), data analysis was carried out, beginning with filtering raw reads output from Illumina Hiseq2500 platform. The sequencing depth for each sample was > 40 million reads. The quality of the reads was checked using the FastQC tool (40). The reads were aligned with Bowtie2 (41) to the hg38 reference genome. The tool coverage bed from BEDTools (42) was used to extract the count per transcript per sample using the annotation files. Differential expression analyses of drug-treated samples against control samples were performed using the DESeq R package (43). DE genes were analyzed using a hypergeometric test and the Benjamini & Hochberg method. Heatmap and hierarchical clustering were done to understand the expression profile based on the value of significantly differentially expressed transcripts.

miRNA-seq: As described (34), data analysis was carried out in the following steps: filtering was done on raw reads output from Illumina Hiseq2500 platform. The sequencing depth for each sample was >10 million reads. The quality of the reads was checked using the FastQC tool (40), and >90% of reads had a Phred score(Q) > 30. Trimming was done using trim_galore (44) to obtain read lengths of 18-25bp. The alignment was performed using Bowtie2 (41), and differentially expressed miRNA was obtained, as mentioned above.

### Integrated Enrichment and Network Analysis of mRNA-miRNA

miRtarvis+ (45, 46) and miRmapper (47) tools were used for studying the interaction between mRNA-miRNA. The miRmapper output was used to find pathways using MIENTURNET (48). miRNA-mRNA network was generated using miRtarvis+ (45, 46). The protein-protein interaction network was created using the STRING database (49). The interacting miRNAs were added manually to the network. However, every miRNA target was verified using TargetScan (50), mirTarBase (51) and miRDB (52).

### Proliferation Study by Lactate Dehydrogenase Assay

The LDH (Lactate Dehydrogenase) release assay was done as described (2, 5). For this assay, 5000 cells were seeded in each well of the 96-well plate in triplicates. After 24 h incubation, cells were induced with 25nM phorbol ester(phorbol 12- myristate 13-acetate; PMA), a known NF-κB activator, for 24h. Treatment with 50nM of ST08 and 500nM IKK-16 was given to PMA-induced MDA-MB-231 cells individually and in combination for 48h. IC50 of IKK-16 in MDA-MB-231 was also evaluated using LDH assay.

### Real-time PCR

As described (34), cDNA was synthesized from 1–2 μg RNA using cDNA synthesis kit from Takara Bio according to the manufacturer’s instructions. Using StepOnePlus™ real-time PCR system from Applied Biosystems and iTaq™ Universal SYBR^®^ Green supermix from Bio-Rad, PCR was set with miR-708a, RELB, MMP1 primers. The sequences for the primers are provided in **Supplementary Figure 5**. The relative level of the target gene from each sample was determined by normalizing it to U6 for miR-708a and GAPDH for MMP1, RELB. All experiments were done in triplicates and repeated at least twice to duplicate results.

## Results

### ST08 Treatment Leads to G2/M Arrest in MCF7 and Induction of Apoptosis

**Figure 1:**
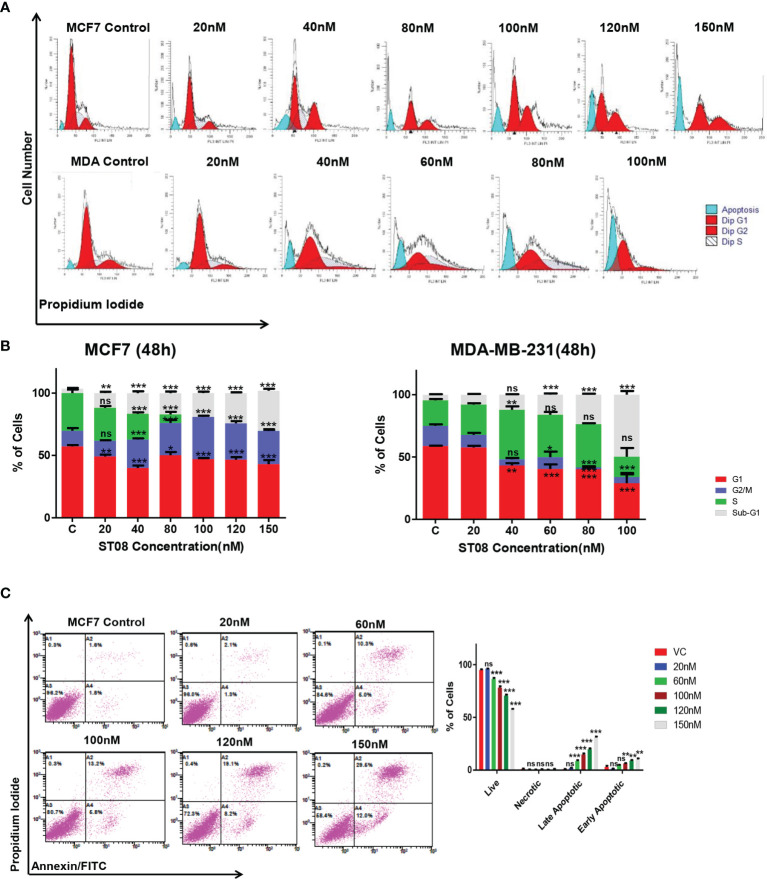
Evaluation of cell cycle progression and cell death modality in breast cancer cells after ST08 treatment: Cell cycle profile **(A)** of 48h ST08 treated MCF7, MDA-MB-231 cells and Quantification of percentage **(B)** of MCF7 and MDA-MB-231 cells in each phase of the cell cycle is depicted as a bar graph of mean ± SEM after 48h treatment of ST08. Each experiment was repeated three times and represented as histograms. Two-way ANOVA test was performed and p-value was calculated between control and ST08 treated groups (*p < 0.05, **p < 0.005, ***p < 0.0001. **(C)** Phosphatidylserine externalisation assay was performed on MCF7 cells after ST08 treatment. Dot plot depicting MCF7 cells treated with ST08 (20,60,100,120nM) for 48h followed by double staining with Annexin-FITC/PIand quantification of cells in different stages. Each experiment was repeated thrice. Two-way ANOVA test was performed and p value was calculated between control and ST08 treated groups (*p < 0.05, **p < 0.005, ***p < 0.0001, ns, not significant).

We have previously reported IC50 of ST08 on MCF7 as 121nM and MDA-MB-231 as 54nM [5]. Flow cytometry analysis was performed to check whether the decrease in cell proliferation was due to cell cycle arrest. MCF7 and MDA-MB-231 cells were treated with increasing concentration of ST08 (20, 40, 80, 100, 120 and 150nM) for 48 h. Both cell lines showed a comparable increase in the G1, sub-G1 population at 40nM ST08; whereas MCF-7 cells showed an increase in G2/M with increasing concentration of ST08 (**Figures 1A, B**). The increase in the sub-G1 population in a concentration-dependent manner upon ST08 treatment indicated cell death. To find out the mechanism by which cells underwent cell death, we performed Annexin-FITC/PI double staining. MCF7 cells, like MDA-MB-231 (5), exhibited an increase in apoptotic cell population in a dose-dependent manner upon ST08 treatment (**Figure 1C**). A negligible population of necrotic cells was observed, suggesting ST08 induced cell death *via* apoptosis.

### ST08 Disrupts the Mitochondrial Membrane Potential

**Figure 2:**
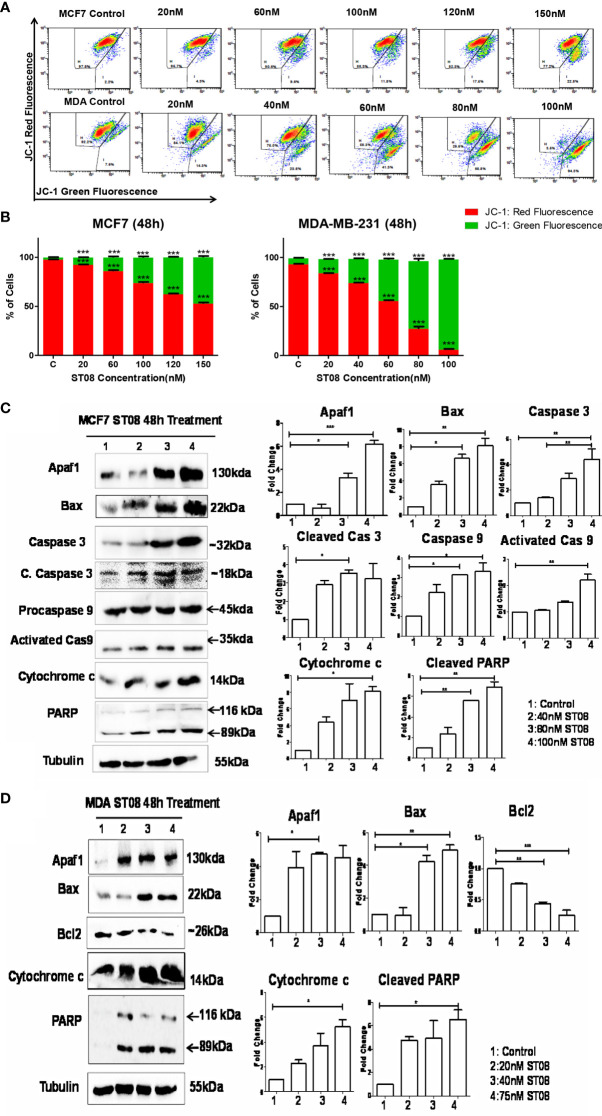
Evaluation of mitochondrial membrane potential(MMP) changes induced in breast cancer cells by JC-1 staining and Assessment of apoptotic protein markers in breast cancer cells treated with ST08: Dot plot depicting 48h treated MCF7, MDA-MB-231 cells by ST08 subjected to JC-1 staining **(A)**. Quantification of high and low MMP **(B)** depicted as a bar graph after 48h treatment of ST08 on MCF7, MDA-MB-231 cells. Each experiment was repeated three times and represented as histograms. Two-way ANOVA test was performed and p-value was calculated between control and ST08 treated groups (*p < 0.05, **p < 0.005, ***p < 0.0001). Western blot analysis of apoptotic markers was done on ST08 treated MCF7 **(C)** and MDA-MB-231 **(D)** cell lysates. Each experiment was done in duplicates, and a representative image is shown for each marker. Quantification was done for each marker and is represented as a bar graph of mean +/- SEM. One sample t-test and one way ANOVA test was performed, and the p-value was calculated between control and ST08 treated groups (*: *p*-value < 0.05, **: *p*-value < 0.005).

JC1, a cationic dye, accumulates in the mitochondria, forming aggregates exhibiting red fluorescence in healthy cells. In apoptotic cells, the mitochondrial membrane is leaky, JC1 cannot form aggregates, and exhibits green fluorescence (53). To check whether a change in mitochondrial membrane potential mediated the apoptotic cell death, JC-1 staining was performed. The flow cytometry analysis (**Figures 2A, B**) showed an increase in the green population upon ST08 treatment in both the cell lines in a dose and time-dependent manner (**Supplementary Figure 1**), indicating mitochondrial damage. No damage to mitochondria was observed in vehicle control. As observed in cell cycle analysis, the effects are similar at equitoxic concentrations in the two cell lines. Thus, the *in vitro* results suggest that ST08 induces apoptosis by altering mitochondrial membrane potential.

### ST08 Induces Intrinsic Pathway of Apoptosis

The mechanism of mitochondrial-mediated apoptosis upon ST08 treatment was unveiled by performing immunoblotting. The loss of mitochondrial membrane potential is a characteristic of the intrinsic apoptotic pathway (54, 55). The concentration-dependent increase in the intrinsic pathway proteins like Apaf1, Caspase 9, Caspase 3, cleavage of PARP, Bax, and cytochrome c (**Figures 2C, D**) was observed in MCF7 breast cancer cells. A similar profile was observed for MDA-MB-231 cells (5). Thus, ST08 induced apoptosis *via* the intrinsic apoptotic pathway. Further, we investigated the impact of ST08 on mouse breast cancer models.

### ST08 Induces Tumor Regression in an EAC Mice Tumor Allograft Model for Breast Cancer

The impact of ST08 *in vivo* was studied using EAC mice tumor allografts. EAC tumor-induced mice were segregated into two groups (Control, ST08 treatment group) with n=5 animals. Ten doses of ST08 (20mg/kg body weight) were given intraperitoneally every alternate day in the treatment group. Throughout the experiment, body weight and tumor size were monitored. A significant reduction in the tumor size was observed after ST08 treatment compared to control (**Figure 3A**). Drug toxicity related to weight loss was monitored by measuring the weight of all the experimental animals. No significant decrease in body weight was observed after the ST08 treatment (**Figure 3B**). Hematoxylin-Eosin staining of the sections from ST08 treated tumors showed a significant reduction in the purple-stained nuclei compared to the control tumor, indicating a reduction in proliferating cells(**Figure 3C**). A large number of proliferating cells in control tumors were observed compared to treated tumors. Also, the cells in the treated tumor tissue showed the presence of fragmented nuclei, indicating apoptosis. To check whether ST08 treatment led to systemic toxicity, both blood and serum were collected for analysis. Serum levels of AST, ALT, and BUN showed no significant difference between the treated and control groups indicating no adverse effects on liver and kidney functions(**Figure 3D**). The hematological parameters, like RBCs and WBCs, were also counted. RBCs levels were maintained, whereas an increase in WBCs was observed (**Figure 3E**). To test whether any morphological and cellular changes were observed in the liver, spleen, and kidney tissue, sections were stained with Haemotoxylin and Eosin. No morphological or cellular changes were observed in the control vs. treated group, indicating no noticeable hepatotoxicity or renal toxicity (**Figure 3C**). Thus, ST08 did not induce any histopathological changes in the animals, indicating no adverse side effects, although a slight increase in WBCs was observed.

**Figure:**
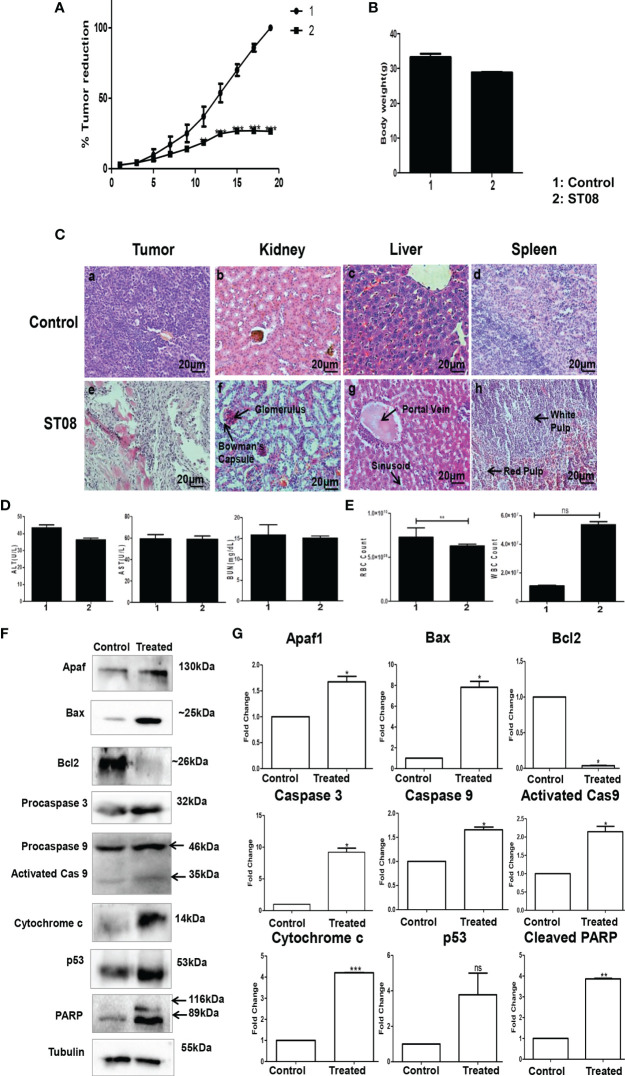
**FIGURE 3** | Evaluation of the effect of ST08 on tumour growth: EAC cells (1 x 10^6^ cells/animal) were injected to induce solid tumours. After the 7^th^ day of injection, i.p injection with ST08 (20mg/kg bd.wt) was started every alternate day throughout the experiment. **(A)** % Tumour volume reduction after ST08 treatment. **(B)** Body weight of animals at the end of the study. **(C)** Histopathological analysis of tumour and organs after ST08 treatment. At the end of the study, tumour tissue and organs were collected and used for histological analysis—representative images of H&E stained sections at 20X magnification of (a) control tumour, (e) ST08 treated tumour (b) control liver, (f) ST08 treated liver (c) control spleen, (g) ST08 treated Spleen (d) control kidney, (h) ST08 treated kidney. **(D)** Blood ALT, AST, Urease test results are plotted as bar graphs. Blood was collected at the end of the study. **(E) **WBC and RBC counts of experimental animals are plotted as bar graphs. **(F) **Effect of ST08 on the expression of apoptotic proteins in the tumour tissue of experimental animals. Tissue lysates were prepared from the dissected tumour samples of post treatments. 40 µg of protein was loaded in SDS-PAGE and checked for apoptotic protein expression by western blotting. **(G)** Quantification was done for each marker and is represented as a bar graph of mean +/- SEM. One sample t-test was performed, and the p-value was calculated between control and ST08 treated groups (*: *p*-value < 0.05, **: *p*-value < 0.005, ***: *p*-value < 0.001, ns, not significant).

### ST08 Activates the Intrinsic Apoptotic Pathway *in vivo*

To delineate the mechanism of cell death *in vivo*, immunoblotting for apoptotic markers was performed. Western blot analysis of protein from treated and untreated tumor tissues was performed. Intrinsic apoptotic markers like Apaf1, Bax, Caspase 3, Caspase 9, PARP, and cytochrome c (proapoptotic) were assessed. All the markers showed a significant increase in their levels except Bcl2 (antiapoptotic), significantly downregulated (**Figures 3F, G**). Also, p53, the genome’s guardian and a known tumor suppressor, showed elevated levels in the ST08 treated tissue. All the experiments show that ST08 activates the intrinsic apoptotic pathway *in vivo*.

### ST08 in Combination With Cisplatin Exhibits Synergistic Cytotoxic Effect

**Figure 4:**
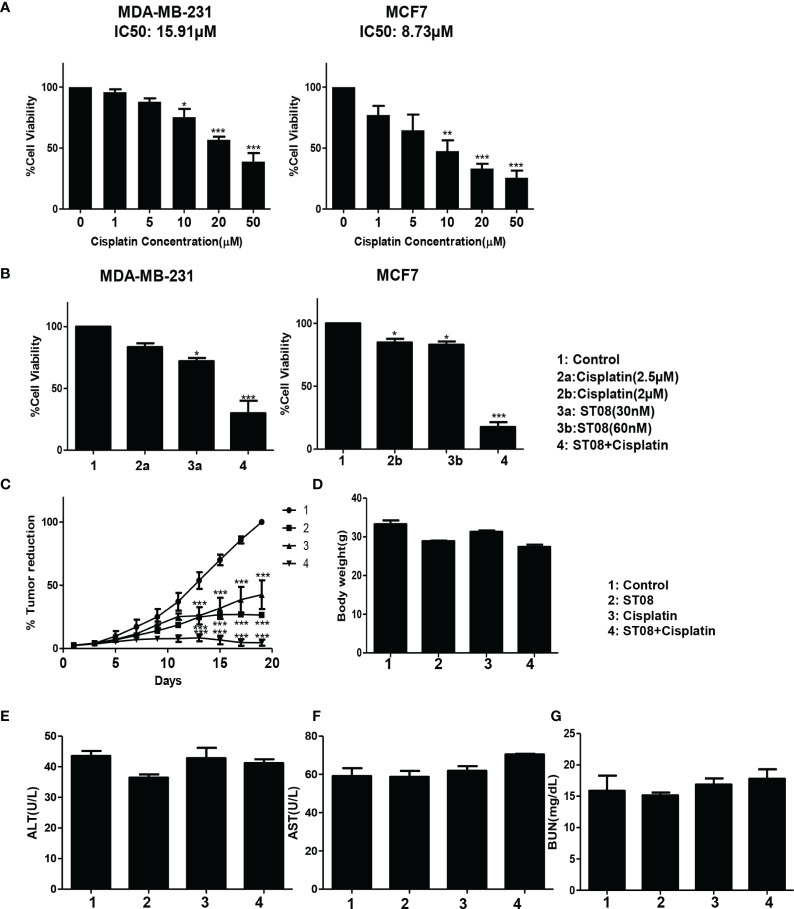
Drug combination study *in vitro* and *in vivo*: Bar graph depicting cell viability upon Cisplatin** (A) **and Combination(ST08+Cisplatin) treatment** (B) **of 48h on breast cancer cell lines as tested by MTT assay. Experiments were performed a minimum of three times, and the bar graph shows mean ± SEM. Two-way ANOVA test was performed and p-value was calculated between control and treated groups (*p < 0.05, **p < 0.005, ***p < 0.0001). EAC cells (1 x 10^6^ cells/animal) were injected to induce solid tumours. After the 7^th^ day of injection, i.p injection with ST08 (10mg/kg bd.wt), Cisplatin(1mg/kg bd.wt), and ST08+Cisplatin was started every alternate day throughout the experiment period. ** (C) **% Tumour volume reduction after ST08, Cisplatin, ST08+Cisplatin treatment. **(D)** Body weight of animals at the end of the study. Blood ALT** (E)**, AST** (F)**, Urease** (G) ** test results are plotted as bar graphs. Blood was collected at the end of the study.

In the clinic, drugs like Cisplatin, Olaparib, Doxorubicin are used for treatment. Cisplatin is also known for drug resistance (56), nephrotoxicity (57–59), Olaparib is associated with hematologic toxicity (60, 61), whereas Doxorubicin is associated with cardiotoxicity (62, 63). We screened all three drugs to check whether ST08 sensitizes the breast cancer cells to their lower dose. IC50 values for each drug on MDA-MB-231, MCF7 cell proliferation were calculated (**Table 2**, **Figure 4A** and **Supplementary Figures 2, 3**). For combinatorial analysis, various concentrations of each drug were used to inhibit cell proliferation (**Supplementary Table 1**). The combination index for MDA-MB-231and MCF7 was calculated using the Chou-Talalay plot (38, 39) for each drug combination as shown in **Table 3** and **Supplementary Figures 2, 3**. Cisplatin showed a synergistic effect with ST08 in both cell lines in a similar concentration range (**Figure 4B**). 30nM ST08+2.5µM Cisplatin for MDA-MB-231 cells and 60nM of ST08+2µM of Cisplatin for MCF7 cells showed inhibition of cell proliferation compared to either drug alone. Similarly, 20nM ST08+1µM Olaparib for MDA-MB-231 cells and 40nM of ST08+5µM of Olaparib for MCF7 cells showed inhibition of cell proliferation compared to either drug alone, whereas 20nM ST08+0.5µM Doxorubicin for MDA-MB-231 cells and 40nM of ST08+10µM Doxorubicin for MCF7 cells showed inhibition of cell proliferation compared to either drug alone. It is important to note that the concentrations used for combination analysis were lower than their respective IC50 values in cell lines. Thus, ST08 can potentiate the effect of Cisplatin, Olaparib, and Doxorubicin by reducing its effective concentration by ~3-fold in both the cell lines.

**Table 2:** IC50 values for Cisplatin, Olaparib, and Doxorubicin in MDA-MB-231, MCF7 cells.

Drug	IC59 for MDA-MB-231 (μM)	IC50 for MCF7 (μM)
Cisplatin	15.91	8.73
Olaprib	11	11.34
Doxorubicin	0.7	8.8

**Table 3:** Combination index(CI) for different drug combinations in MDA-MB-231, MCF7 cells.

ST08+Drug	CI for MDA-MB-231	CI for MCF7
Cisplatin	Synerism (30nM ST08+2.5 μM)	Synergism (60nM ST08+ 2μM)
Olaprib	Synerism (20nM ST08+1μM)	Synergism (40nM ST08+5 μM)
Doxorubicin	Additive (20nM ST08+0.5μM)	Additive (40nM ST08+10 μM)

### ST08 Combination With Cisplatin Reduces Tumor Growth Significantly in an EAC Mice Tumor Allograft Models for Breast Cancer

After performing *in vitro* combination treatment experiments, *in vivo* studies were performed. As described (2), once the tumor was developed ~200mm3, the intraperitoneal alternate drug treatment of 10 doses was initiated. Animals were segregated into 4 groups(each group n=5; control, ST08 20mg/kg bd wt, Cisplatin 1mg/kg bd wt, ST08 (10mg/kg bd wt)+Cisplatin (1mg/kg bd wt). Bodyweight and tumor volumes were monitored throughout the experimental time. It can be seen that the combination treatment gave the best tumor reduction rather than individual drugs in comparison to the untreated control group (**Figure 4C**). Also, there was no significant change in the body weights of all the groups (**Figure 4D**), indicating no major side effects.

### Combination Treatment Induces Minimal Side Effects With the Least Toxicity

Although no change in body weight was observed, to check if the drug had any adverse effect on the liver and kidney, Serum ALT, AST, and BUN assays were performed. As shown in **Figures 4E-G**, ALT, AST, and BUN levels were within the normal range in all the treatment groups, indicating that ST08 can be used with cisplatin for combination therapy resulting in tumor reduction, minimal side effects, and least toxicity.

Although the drugs show promising results in animal models, only a few make it to the clinic. Before taking the drug to the clinic, it would be necessary to check if it can induce global cellular changes leading to toxicity or drug resistance. One of the most widely used tools to investigate drug effects is by measuring changes in the expression of genes using RNA-seq. We have performed miRNA and RNA seq of control and ST08. 

### Transcriptomic Analysis of Breast Cancer Cells Upon ST08 Treatment Using RNA-seq

Differential gene expression analysis was performed with MDA-MB-231 breast cancer cells treated with 75 nM ST08 for 48h. The gene expression profiling was performed using RNA-seq. The data used for analysis is from three biological replicates. More than 20 million reads were generated, with ~80-83% alignment for all the RNA-seq data using the reference genome(hg38) (**Supplementary Table 2** and **Supplementary Figures 4 A, B**). A total of 461 genes in MDA-MB-231 were differentially expressed(DE) (Log2 Fold Change > 0.75, P-value < 0.05), of which 42.82% were upregulated, and 57.18% were downregulated (**Supplementary Figure 5**).

**Figure 5:**
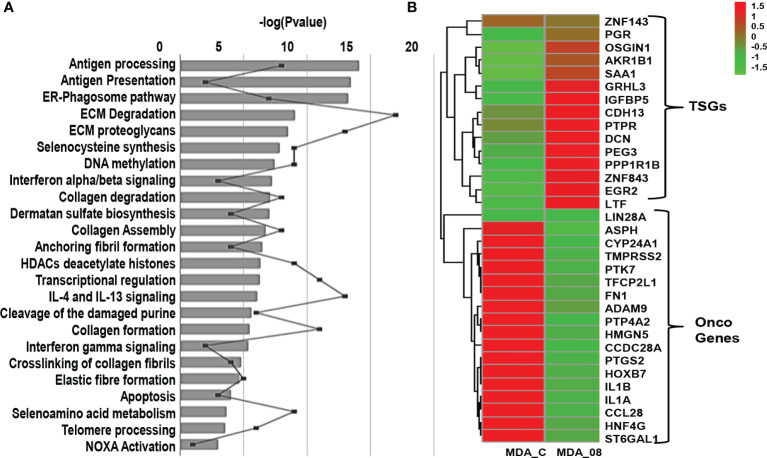
Transcriptome profile of breast cancer cells upon ST08 treatment: **(A)** Pathways regulated in MDA-MB-231 TNBC cells upon ST08 treatment. **(B)** Heatmap of DE tumor suppressor and oncogenes genes in MDA-MB-231upon ST08 treatment.

Further, to analyze the pathways which were altered, DE genes were subjected to pathway analysis. The pathways altered by ST08 related to immune response were ER-Phagosome pathway, Antigen processing-Cross presentation Interferon gamma signaling, Endosomal/Vacuolar pathway, Antigen Presentation: Folding, assembly and peptide loading of class I MHC pathways. In addition, the chromatin modifiers HDACs pathway, ECM degradation, and Collagen degradation were also enriched (**Figure 5A**).

Since cancer is driven by an imbalance of oncogene and tumor suppressor gene(TSG) expression, we analyzed the significant DE genes for TSGs and oncogenes. For TSGs, we used the TSG database (64)and selected TSGs associated with breast invasive carcinoma samples, and for oncogenes, we used the Oncogene database (65). 589 TSGs were found in the TSG database for breast adenocarcinoma (BRCA) and 803 oncogenes in the Oncogene database. The percentage of upregulated TSGs and downregulated oncogenes were calculated using the significant DE genes. 50% TSGs were upregulated in MDA-MB-231 cells upon ST08 treatment, whereas 40-46% oncogenes were downregulated. Heatmaps were plotted for these TSGs and oncogenes, indicating the regulation of DE TSGs and oncogenes after ST08 treatment in MDA-MB-231 cells (**Figure 5B**).

### miRNA-seq Analysis of Breast Cancer Cells Upon ST08 Treatment

The MDA-MB-231 cells were treated with ST08 as mentioned above, and miRNA-seq was performed. Around 23 million reads were obtained from biological replicates. 82-97% alignment was achieved with processed reads using the reference genome(hg38) (**Supplementary Table 2** and **Supplementary Figures 4C, D**). Differentially expressed (DE) miRNAs (Log2 Fold Change > 0.75, P-value < 0.05) upon ST08 treatment were identified (**Supplementary Figure 6**).

Similar to RNA-seq, a tumor suppressor and oncomiR analysis were performed for DE miRNAs. A validated list of 39 TS miRs and 17 oncomiRs specific for breast cancer was adapted from (34) (**Supplementary Table 3**). Among them, 35% TS miRNAs (miR-543, miR-26a, miR-708, miR-26b, miR-340,miR-204,miR-296) were upregulated and 21% of oncogenic miRNAs (miR-135b,miR-203,miR-21,miR-24) were downregulated in MDA-MB-231 cells treated with ST08 (**Supplementary File 2- List of DE miRNAs after ST08 treatment**). Interestingly, miR-129-2-3p (66, 67), hsa-miR-223 (68), and miR-372-3p (69), which inhibit proliferation and biological behavior of TNBC cells, were significantly upregulated. We also analyzed miRs that regulate drug resistance in breast cancer (70) miRs like miR-125b, miR-663, miR-221, and miR-203 were downregulated in MDA-MB-231 cells upon drug treatment.

### Integrated mRNA-miRNA Seq Analysis of Breast Cancer Cells Upon ST08 Treatment

**Figure 6:**
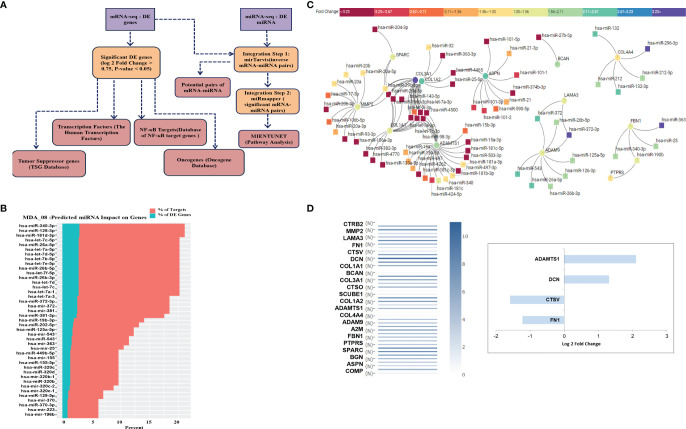
Integrated analysis of miRNA-mRNA and Network analysis: **(A)** Flowchart showing the integration of miRNA-mRNA seq data. **(B)** DE expressed miRNA-mRNA data from MDA-MB-231 cells after ST08 treatment was integrated using miRmapper. **(C)** miRNA-mRNA network for ECM organization and degradation, regulated by ST08 in MDA-MB-231 cells. **(D)** Drug-induced changes were either upregulated/downregulated for expression in the tumour and reverted to normal expression post ST08 treatment.

We have established an effective pipeline for analyzing the whole transcriptome data. The flowchart (**Figure 6A**) gives the details of the software used at each step. The DE miRNAs with log fold change > 1 and mRNA DE genes with log fold change > 1.5, P-value < 0.05 were given as input for miRTarVis (45, 46) miRTarVis returned inversely related pairs of miRNA-mRNA, which was used as input for miRmapper. Using miRmapper, we obtained the list of miRNAs that regulated the maximum number of DE genes. A total of 76 miRNAs from MDA-MB-231 were obtained as output which regulated the DE mRNAs. The top 40 miRNAs have been represented as bar graphs (**Figure 6B**). As shown in **Figures 6A, B**, it can be seen that hsa-miR-340-3p interacts with a maximum of 2.85% DE genes in MDA-MB-231 treated with ST08. We subjected the output of miRmapper to pathway analysis using MIENTURNET. The pathways enriched in ST08 treated MDA-MB-231 were metabolic pathways, signal transduction, immune system, metabolism of proteins, post-translational protein modification.

### miRNA-mRNA Network Analysis

We built a miRNA-mRNA network for uniquely regulated pathways by ST08. As observed, ST08 altered miRNA and mRNA expression, some of which may be regulated by miRNA. One of the pathways of importance identified was Extracellular matrix related pathways; this coincided with our previous observation, migrastatic properties of ST08 in MDA-MB-231 cells. 23 unique genes were enriched in Extracellular matrix related pathways of MDA-MB-231. 23 ECM genes and DE miRNA were given as input for miRTarvis. miRtarvis generated a miRNA-mRNA network of 106 interactions for MDA-MB-231 (**Figure 6C**).

To understand the alteration in ECM genes in the context of its normal vs. tumour expression in patient samples, we checked the expression of ECM genes in breast normal and tumor samples using GEPIA (**Figure 6D**). Interestingly, the ECM genes upregulated in patient tumor samples were downregulated in ST08 treated cells and vice-versa. Alterations in ECM are known to promote cancer metastasis (71, 72), and MDA-MB-231 is a model cell line to study breast cancer metastasis (2, 5). Most of the genes in the network restored normal expression (compared to the tumor) after ST08 treatment, indicating one of the mechanisms by which ST08 controls cell proliferation and migration. For example, MDA-MB-231 cells treated with ST08 showed upregulation of genes like DCN and ADAMTS1 and downregulation of CSTV and FN1, indicating restored normal expression as in normal breast tissues and favorable outcome (**Figure 6D**). Some of the changes in gene expression upon ST08 can be attributed to an alteration in miRNA. As shown in **Figure 6C**, Bioinformatic analysis identified miR-15a, miR-181, miR-497, miR-4262, miR-424, miR-503 that have binding sites in 3’UTR of ADAMTS1, downregulated in MDA-MB-231 after ST08 treatment, and ADAMTS1, a tumor suppressor upregulated. Similarly, ASPN, a tumor suppressor, is upregulated, and miRNA targeting is downregulated. On the contrary, we observe upregulated oncogene like COL1A1, COL3A1, and downregulated miRNA, which did not correlate with downregulated collagen *in vivo* in treated tumour samples (data not shown).

To validate the results obtained from miRNA-mRNA analysis in breast cancer cells, we selected the NF-κB pathway, as it is known to be overexpressed in breast tumour samples (73) and TNBC has high NF-κB compared to ER+ve cancers (74). We validated NF-κB and its targets in TNBC cell line MDA-MB231 cells.

### ST08 Targets NF-κB in Breast Cancer Cells

**Figure 7:**
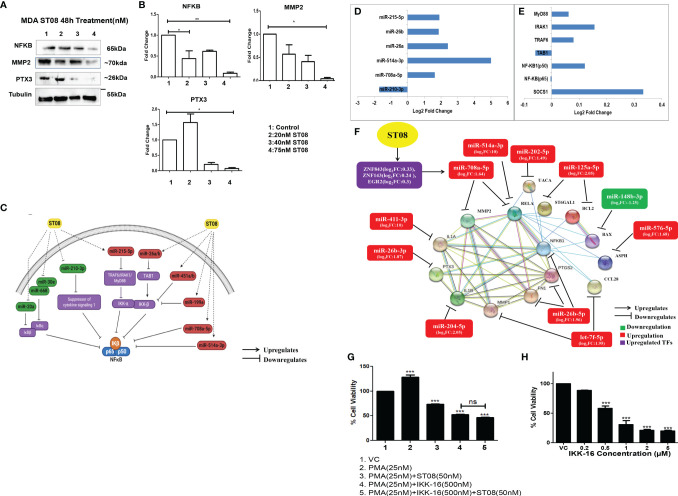
NF-κBregulation in MDA-MB-231 cells upon ST08 treatment: **(A)** Western blot analysis NF-κB protein and its targets was done on ST08 treated MDA-MB-231 cell lysates. Each experiment was done in duplicates, and a representative image is shown for each marker. Quantification was done for each marker** (B) **and is represented as a bar graph of mean +/- SEM. One sample t-test and one way ANOVA test was performed, and the p-value was calculated between control, and ST08 treated groups (*: *p*-value < 0.05, **: *p*-value < 0.005). **(C)** ST08 induced upregulation of miRs like miR-215-5p,miR-26a/b,miR-451a/b,miR-199a,miR-708a-5p and miR-514a-3p which directly or indirectly inhibited NF-κB. Whereas miRs like miR-20a, miR-668, miR-30e, miR-210-3p were downregulated, which upregulated IKB, SOCS1, and indirectly downregulated NF-κB.Bar graph depicting log2 Fold change of miRNA** (D) **and mRNA** (E) **involved in NF-κB pathway. **(F)** miRNA-mRNA regulatory network of NF-κB pathway upon ST08 treatment in MDA-MB-231 cells. ST08 regulates TFs like ZNF843, ZNF143, and EGF3 to regulate the expression of miR-708a-5p. miR-708a-5p,miR-514a-3p regulates NF-κB. ST08 also regulates expression of other miRs like miR-411-3p,miR-26b-3p/5p, miR-205-5p,let-7f-5p, miR-576-5p, miR-148b-3p,miR-125a-5p and miR-202-5p. These miRs regulate the expression of their targets, as shown in the network. **(G)** Effect of ST08 and IKK-16 on PMA-induced proliferation in MDA-MB-231 cells as tested by LDH assay. **(H)** Bar graph depicting cell viability upon IKK-16 treatment for 48h on MDA-MB-231 breast cancer cell line as tested by LDH assay. Experiments were performed a minimum of three times, and the bar graph shows mean ± SEM. One-way ANOVA test was performed and p-value was calculated between control and treated groups (*p < 0.05, **p < 0.005, ***p < 0.0001, ns, not significant).

Integrated miRNA-mRNA analysis of ST08 treated MDA-MB-231 returned top pathways as altered ECM and metabolism. All the previous analysis of NF-κB was based on protein expression. We also had observed downregulation of NF-κB (RElA-p 65) at the protein level. Therefore, we checked for NF-κB levels at the transcript. No significant change was observed at the transcript level (**Figure 7E** and **Supplementary Figure 7**). In contrast, a significant decrease in protein level was observed at a 75nM concentration of ST08 (**Figures 7A, B**), suggesting regulation of translation of NF-κB mRNA. We also checked for the expression levels of the p50 subunit of NF-κBby RT-PCR. RNA-seq and RT-PCR showed p50 upregulation in ST08 treated MDA-MB231 (**Supplementary Figure 7**). One of the known translation regulators is miRNA. To identify miRs against NF-κB, we utilized TargetScan (75). We identified several miRNAs against the NF-κB pathway, differentially expressed in ST08 treated samples. One of the miRs, miR-708a-5p, was significantly upregulated and may regulate NF-κB. We also obtained differentially expressed transcription factors that can bind to the promoter of the miRNA and regulate gene expression. We found that TFs like ZNF843, ZNF143, EGR2 were upregulated (**Figure 7F**), and they most probably regulated miR-708-5p expression.

To understand the regulation of NF-κB and its regulators by miRNA, we manually introduced the differentially expressed miRNA to the known NF-κB pathway (76). It is very well known that NF-κB is bound to IKβ, and IKK guides IKβ phosphorylation/ubiquitination (77), leading to NF-κB entry to the nucleus. Therefore, we cataloged miRNAs that regulate IKK and IKβ using miRNA-seq data (**Supplementary File 2-DE miRNAs in MDA-MB-231 upon ST08 treatment**). miR-215-5p was upregulated upon ST08 treatment, which might block TRAF6/IRAK1/MyD88 and, therefore, no phosphorylation of its downstream target IKKα. Similarly, miR-26a/b can bind to the 3’UTR of TAB1 and downregulate/degrade the RNA, leading to the absence of activity of IKKβ. miR-451a/b,miR-199a, which can also bind to 3’UTR of IKKβ, were downregulated, and miR-708a-5p and miR-514a-3p that have the binding site in 3’UTR of NF-κB were upregulated in MDA-MB-231 cells treated with ST08. The levels of miR-20a, miR-668, miR-30e that target IKBα/β and miR-210-3p, which can bind to 3’UTR of SOCS1, were downregulated SOCS1 was upregulated, which blocks IKβ phosphorylation in MDA-MB-231 cells treated with ST08 (**Figure 7C**). ST08 induced downregulation of NF-κB protein can be attributed to alterations in the miRNA levels, targeting different genes in the NF-κB pathway. (**Figures 7C–E**).

Integrated miRNA and mRNA analysis of the NF-κB pathway of MDA-MB231 cells treated with ST08 suggested that ST08 altered miRNA to regulate the NF-κB pathway. Since NF-κB was downregulated, we checked for the downstream targets of NF-κB in the DE gene list. We obtained the NKFB target, which consists of 1667 distinct genes (20). Out of these, 31 targets were found differentially expressed in the MDA-MB-231 gene list upon ST08 treatment. Oncogenic NF-κB targets like PTGS2 (78), CCL28 (79), IL1A (80), FN1 (81),ASPH (82), ST6GAL1 (83), IL1B (84), MMP1 (2, 85), PTX3 (86–89), Bcl2 (90, 91) were significantly downregulated in TNBC MDA-MB-231 cells upon ST08 treatment. One of the targets in this list was MMP2; however, mRNA levels of MMP2 were significantly upregulated (**Supplementary File 1-DE** mRNAs in MDA-MB-231 upon ST08 treatment). Our previous study showed that ST08 inhibits migration (5), and hence we checked protein levels. Western blotting with MMP2 showed significant downregulation of protein (**Figures 7A, B**). Interestingly, hsa-miR-708a-5p also targets MMP2 (92, 93), suggesting miRNA mediated translation inhibition of MMP2 protein. We also validated the expression of hsa-miR-708a-5p upregulation in treated cells by RT-PCR (**Supplementary Figure 5**).

We also validated some of the DE genes downstream of NF-κB (20) like MMP1, PTX3, Bcl2, and Bax. We have previously shown downregulation of MMP1 protein in MDA-MB231 cells (5). We show here by RT-PCR of MMP1 (**Supplementary Figure 7C**) its downregulation and by western blotting PTX3, Bcl2 (**Figure 2D**) downregulation, whereas Bax (**Figure 2D**) upregulation upon ST08 treatment.

We have generated an interaction network for the NF-κB pathway involving miRNA-mRNA and proteins regulated by ST08 in MDA-MB-231 cells (**Figure 7D**). The protein-protein interaction network was generated using the STRING database (49), and miRNAs and TFs were manually added to the network. All the targets of NF-κB except Bax were downregulated in our dataset. One of the mechanisms by which ST08 could have downregulated gene expression of the downstream NF-κB genes is *via* regulating miRNA. Therefore we also checked for miRNAs against targets of NF-κB. We found significant differentially expressed miRNAs that were upregulated and respective genes downregulated (**Figure 7F**), suggesting ST08 mediated cell proliferation and migration inhibition by altering both miRNA and mRNA.

To further establish that ST08 mediated its anticancer activity *via* NF-κB, we designed a proliferation assay with a known activator (phorbol 12- myristate 13-acetate; PMA) and inhibitor(IKK-16) of NF-κB. Activation of the NF-κB pathway by phorbol ester(phorbol 12- myristate 13-acetate;PMA) is very well documented (94–96). PMA activates protein kinase C (PKC) isozymes by binding to diacylglycerol (DAG) receptor sites in the N-terminus of these proteins, which lead to the activation of NF-κB, a major transcription factor (97). We evaluated the antiproliferative potential of ST08 on PMA-induced invasion of MDA-MB-231 cells. 48h treatment of 50nM ST08 on PMA-induced (25nM, 24h) MDA-MB-231 cells led to a significant reduction in cell viability (**Figure 7G**). We used IKK-16, a known NF-κB inhibitor (98, 99), as a positive control for comparing ST08’s potential for inhibiting NF-κB. The IC50 of IKK-16 for MDA-MB-231 was evaluated as 480 nM after 48h treatment (**Figure 7H**). 500nM IKK-16 was added to PMA-induced (25nM,24h) MDA-MB-231 cells which led to a reduction in cell viability comparable to ST08 (**Figure 7H**). We further used a combination of ST08 and IKK-16 to check its effect on PMA-induced proliferation. No significant difference in inhibition was observed between IKK-16 and ST08 vs. IKK-16 alone, indicating that ST08 mediated inhibition of proliferation *via* the NF-κB pathway, further establishing that one of the pathways altered by ST08 is the NF-κB pathway.

## Discussion

In the present study, we have elucidated the cell death mechanism induced by ST08, a previously reported curcumin derivative. Interestingly, integrated miRNA –mRNA analysis revealed ST08 mediated regulation of NFKB and its downstream targets. Importantly, we observed that ST08 (20mg/kg b.wt) reduced tumor burden in EAC mice tumor models. In combination with cisplatin (1mg/kg b.wt), ST08(10mg/kg b.wt) showed drastic tumor reduction in mice models with no apparent liver and renal toxicity. Additionally, integrated transcriptome(miRNA-mRNA) analysis revealed mRNA and miRNA players in ECM regulation in MDA-MB-231 cells.

Breast cancer is known for its multifactorial and aggressive nature in advanced stages. Due to the high metastatic rate, multidrug resistance, and relapse, newer drugs are being explored. However, chemotherapy drugs must show effectiveness with the least possible organ toxicity and side effects. Thus, plant-based therapeutics have been explored. Curcumin is a plant-based therapeutic with multitudinal effects reported in various diseases, including cancer. However, multiple limitations of curcumin hinder its usage, and thus, several curcumin derivatives have been explored. However, only the ST series derivatives viz ST03 (5), ST06 (100), ST08 (5), and ST09 (2) have shown their potency in the nanomolar range. All the members of this series have exhibited their potential to inhibit tumor growth with negligible organ toxicity and side effects.

A drug can induce cell death in multiple ways- apoptosis being the most vital. The two major apoptotic pathways are intrinsic(mitochondrial-mediated) and extrinsic(death receptor-mediated) (5, 101). ST08 induced an intrinsic apoptotic pathway. We have previously reported that ST08 induces cytotoxicity in the MDA-MB-231 breast cancer cell line (5). This study shows that ST08 altered mitochondrial membrane potential and activation of Caspase 9 and Caspase 3 to induce cell death invitro. Also, ST08 reduced tumor burden in mouse EAC model by activation of Caspase 3 protein and apoptosis.

To develop a chemotherapeutic drug with potent anticancer activity, the drug must exhibit negligible organ toxicity and minimal side effects. ST08, like ST09 (2), showed minimal toxicity related to body weight, minimal organ toxicity in liver, kidney, and spleen, and reduced the number of proliferating cells in tumor tissue.

ST08, a curcumin derivative, synergizes with cisplatin, reduces the effective dose of cisplatin, and reduces tumor burden. The *in vivo* effect of the drug might be synergistic or additive and needs further evaluation. Cisplatin, an alkylating agent, is well known for nephrotoxicity (57, 59), and drug resistance (102) limits its usage. Cisplatin is also known to induce apoptosis in MCF7, MDA-MB-231 cells (103–105). In this context, the parent compound curcumin sensitized breast cancer cells to cisplatin (106, 107). Thus, a combination of ST08 and low dose cisplatin might be an effective strategy for inducing apoptosis in cancer cells.

ST08 induced changes in the gene expression were evaluated using RNA and miRNA sequencing. ST08 treated MDA-MB-231 cells showed upregulation of TSG like GPX3, OSGIN1, CLU, CDH13. GPX3, a ROS regulator, is a known TSG in breast cancer (108). Docosahexaenoic acid-induced cell death in Breast cancer cells by upregulating OSGIN1 expression *via* PI3K/Akt/Nrf2 signaling pathway (109). CDH13 is a known tumor suppressor gene, and its promoter is methylated in breast cancer patients (110, 111) and other cancers as well (112, 113). ST08 induced the expression of CDH13. ST08 treatment led to downregulation of oncogenes like UACA, PDE4D, HMGN5. HMGN5 promotes the proliferation and invasion of breast cancer cells, and its knockdown increases apoptosis in breast cancer cells (114). PDE4D overexpression induces colony formation, cell proliferation, and anchorage−independent growth in colorectal cancer cells (115). In breast cancer patients treated with tamoxifen, PDE4D overexpression is associated with worse survival. In addition, PDE4D is known to inhibit cAMP/ER stress/p38-JNK signaling and apoptosis in tamoxifen-resistant ER-positive Breast Cancer cells (116). UACA is an oncogene known to induce apoptosis resistance in cancer (117). UACA expression is regulated by p53 activation and/or inhibition of NF-κB activity (118). Coincidently, ST08 upregulated p53 and downregulated NF-κB.

ST08 regulated miRNA levels in MDA-MB-231 cells miR-668 is crucial in breast cancer’s radiosensitivity and regulates IκBα, a tumor-suppressor, and an NF-κB inhibitor (119). miR-668 downregulation in breast cancer cells by ST08 might restore radiosensitivity. miR-19b-3p regulates PIK3CA and could reverse saracatinib resistance in saracatinib-resistant breast cancer cells. ST08 upregulated miR-19b-3p expression in breast cancer cells, making them targetable by drugs like saracatinib, reinstating the potential of ST08 as a good candidate for combination therapy. On the other hand,miR-155-5p is known for its oncogenic role in breast cancer (120–122) and was upregulated in MDA-MB-231 cells upon ST08 treatment. miR-155 upregulation has been utilized in predicting response to PARP1 inhibitors in the clinical setting (123). ST08 in combination with PARP1 inhibitor might overcome any resistance to ST08 due to elevated miR-155.

We built a miRNA-mRNA network of pathways for understanding gene regulation by miRNAs. miRNA-mRNA data integration using miRmapper showed that miR-340 regulates 2.85% DE genes in MDA-MB-231 cells. miR-340 is a known TS miR in breast cancer and regulates metastasis in TNBC cells by inhibiting EZH2. We got an intricate network for ECM regulation in MDA-MB-231. GEPIA analysis of the genes in the network helped analyze the expression of genes in tumor samples compared to normal samples. ST08 restored the expression of FN1, CTSV, DCN, ADAMTS1 in MDA-MB-231 cells. Decorin(DCN) is representative of small leucine-rich proteoglycans (SLRPs) in the extracellular matrix (ECM). DCN upregulation can predict a good prognosis in breast cancer patients (124, 125). ADAMTS1 downregulation can stimulate migration and invasion in breast tumors (126). ST08 restored normal expression of ADAMTS1 most probably by downregulating miRs, miR-181 (127) and miR-4262 (128), targeting ADAMTS1 in TNBC cells.

We found that ST08 regulates miRNA to control the expression of target mRNA both at transcriptional and translational levels. Pathway enrichment analysis of integrated data showed enrichment of translational block, which can be attributed to ST08 induced miRNA regulation. miR-708-5p is a tumor suppressor known to regulate NF-κB subunit p-65, the master regulator of an array of genes (20) and MMP2. Additionally, PTX3 is exclusively expressed in basal-like breast cancer *via* PI3K-AKT- NF-κB signaling and promotes stem-cell-like traits (86–89). ST08 mediated downregulation of PTX3 at protein levels could be attributed to low NF-κB and miR-26b-3p expression. Similarly, MMP1, MMP2, and Bcl2 protein levels were reduced by ST08 treatment, downstream targets of NF-κB, and correlated with high levels of let-7f-5p, miR-708-5p, and miR-125a-5p expression. In contrast, Bax levels were upregulated by ST08, which correlated with the downregulation of miR-148b-3p. MMP2 (2, 85) and MMP1 (2, 85) are key players in breast cancer proliferation, metastasis, and invasion. Whereas, Bax (129, 130) and Bcl2 (90, 91) regulate apoptosis. Interestingly, the transcription factors ZNF843, ZNF1, and EGR3, having binding sites on the promoter of miR-708-5p, were upregulated by ST08. Thus, ST08 regulates apoptosis, proliferation, metastasis, and invasion by regulating TFs like NF-κB and its downstream targets by modulating protein levels through the action of miRs or downregulation or upregulation of mRNA.

In MDA-MB-231, oncogenic miR-203 was downregulated on ST08 treatment. miR-203 is also responsible for Cisplatin resistance *via* SOCS3 upregulation (131). The observation that ST08+Cisplatin reduced tumor burden in EAC mice tumor model significantly with least liver and kidney toxicity could be through modulation of miR-203. Apart from that Tumor suppressor miRs like miR-26a/b,miR-708, miR-340,miR-204,miR-296,miR-129-2-3p, miR-223 and miR-372-3p which play a crucial role in EMT and cell migration, were upregulated. miR-26a is downregulated in TNBC cells and tumors. On overexpression, it downregulates the expression of metadherin (132) and MCL-1 (133). miR-26b negatively regulated DEPDC1 expression and upregulated FOXM1 expression (134). miR-708 is another promising metastasis suppressor for TNBC. Downregulation of miR-708 elevates intracellular Ca2+ levels *via* neuronatin which promotes migration (135, 136). Thus, multilayered gold nanoparticles carrying miR-708 have been synthesized, which reduces lung metastasis (134, 135). miR-340 is another player in TNBC which exhibits tumor suppressor activity by inhibiting EZH2 (137). miR-204-5p regulates metastasis by directly controlling the expression of PIK3CB and downstream PI3K/Akt signaling activities (138).

ST08 regulated NF-κB levels could be attributed to miRNA regulation of NF-κB translation. NF-κB is known to be regulated by miRNAs like miR-506 and miR-520/373 family (139) and *via* ubiquitin ligases that regulate the NF-κB degradation pathway (115, 140). In this study, we found that miR-708a-5p might have a role in the regulation of NF-κB. Direct targets of NF-κB could be downregulated due to a decrease in NF-κB levels or by ST08 induced miRNA regulation. NF-κB regulates cancer progression by controlling cell cycle, apoptosis, survival, migration, proliferation, cellular metabolism, angiogenesis, therapy resistance, immunosuppression, metastasis, inflammation, and epigenetic alterations (77). NF-κB is one of the downstream targets of curcumin, and its role has been established in carcinogenesis (22–26).

The integrated analysis of miRNA-mRNA led to the identification of critical pathways altered in MDA-MB-231 on ST08 treatment. ST08 altered the NF-κB pathway and downstream targets and synergized with cisplatin, making it a promising chemotherapeutic agent.

## Conclusion

For the first time, we report an integrated miRNA-mRNA analysis in breast cancer cells upon ST08 treatment, which revealed NF- κB as one of the targets.ST08 regulated both upstream and downstream targets of NF-κB. It induced the intrinsic pathway of apoptosis. This study documents the pleiotropic effect of ST08 against highly metastatic, recurrent, and invasive breast cancer by modulating both miRNA and mRNA. ST08 also sensitized breast cancer cells to cisplatin. ST08 alone or in combination can be developed as a potential drug for cancer therapy.

## Data Availability Statement

The data presented in the study are deposited in the BioProject NCBI repository https://www.ncbi.nlm.nih.gov/, accession number PRJNA794262.

## Ethics Statement

The animal study was reviewed and approved by 1994/GO/ReBi/S/17/CPCSEA.

## Author Contributions

SN and BC conceived the idea, designed the experiments, analyzed the data, and wrote the manuscript. SN performed experiments on breast cancer cells. SN and RM performed animal experiments. SN and SD performed the bioinformatics analysis. FR performed combination studies with Doxorubicin and Olaparib. All authors reviewed the manuscript. All authors read and approved the final manuscript.

## Funding

This work was supported by grants from the Department of Science and Technology (SR/FST/LSI-536/2012) and the Department of Biotechnology (BT/PR13458/COE/34/33/2015). SN was supported by DST-INSPIRE (Ref. no. IF140949/2015, Innovation in Science Pursuit for Inspired Research, Dept. of Science and Technology, Govt. of India) Funding from IT,ST and BT, GoK.

## Conflict of Interest

The authors declare that the research was conducted in the absence of any commercial or financial relationships that could be construed as a potential conflict of interest.

## Publisher’s Note

All claims expressed in this article are solely those of the authors and do not necessarily represent those of their affiliated organizations, or those of the publisher, the editors and the reviewers. Any product that may be evaluated in this article, or claim that may be made by its manufacturer, is not guaranteed or endorsed by the publisher.
