# Supplementary figures and images for: ST08 Altered NF-κB Pathway in Breast Cancer Cells In Vitro as Revealed by miRNA-mRNA Analysis and Enhanced the Effect of Cisplatin on Tumour Reduction in EAC Mouse Model
Source: Front Oncol. 2022 May 9;12:835027. doi: 10.3389/fonc.2022.835027 (PMC9125255; doi:10.3389/fonc.2022.835027)

| Structure of ST08                                                                 | Characteristics | MCF7 | MDA-MB-231 |
|-----------------------------------------------------------------------------------|-----------------|------|------------|
| 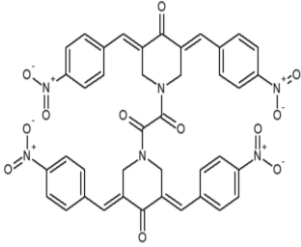 |                 |      |            |
|                                                                                   |                 |      |            |
|                                                                                   |                 |      |            |
|                                                                                   |                 |      |            |

Table 1

Supplement: Supplementary file 1 [file Table_1.pdf]
